# Supplementary material for: Assessment of Dietary Sodium Intake Using the Scored Salt Questionnaire in Autosomal Dominant Polycystic Kidney Disease
Source: Nutrients. 2020 Nov 2;12(11):3376. doi: 10.3390/nu12113376 (PMC7692491; doi:10.3390/nu12113376)
Supplement: Supplementary file 1 [file nutrients-12-03376-s001.pdf]

*Supplementary Material*

**Table S1.** Characteristics of the study population subdivided according to the completeness of the urine collection.

| Parameter | Complete 24-hr Urine Collection (*n*=75) | Under-Collection of 24-hr Urine (*n*=21) | Over-collection of 24-hr Urine (*n*=13) |
|---|---|---|---|
| Age (years) | 44.6±11.5 | 45.1±9.8 | 39.8±10.8 |
| Gender (F:M) (%) | 53:47 | 81:19* | 38:62 |
| Height (m) | 1.71±0.09 | 1.65±0.10* | 1.73±0.08 |
| Weight (kg) | 76.1±15.1 | 82.1±21.3 | 73.9±14.1 |
| BMI (kg/m$^2$) | 25.9±3.8 | 30.1±6.2* | 24.5±3.5 |
| Systolic BP (mm Hg) | 133±14 | 134±15 | 131±15 |
| Diastolic BP (mm Hg) | 85±11 | 86±12 | 81±8 |
| Serum Cr (mmol/L) | 91 (70-123) | 88 (61-105) | 88 (78-104) |
| eGFR (mL/min/1.73m$^2$) | 75 (53-105) | 80 (55-109) | 89 (75-97) |
| 24-hr urine Na (mmol/day) | 132 (112-172) | 99 (86-151) | 183 (128-261)* |
| 24-hr urine Cr (mmol/day) | 13.1±3.4 | 9.1±2.9* | 17.7±3.4* |

Abbreviations: Na, sodium; BMI, body mass index; BP, blood pressure; Cr, creatinine; eGFR, estimated glomerular filtration calculated using the Chronic Kidney Disease Epidemiology Collaboration equation. Data expressed as mean±SD with exception of serum Cr, eGFR and 24-hr urine Na which are presented as median with inter-quartile range; *$p < 0.05$ compared to the Complete Urine Collection group.

**Table S2.** Median absolute values of each of the nine sodium-rich food types from the SSQ.

| Sodium-Rich Food Groups | SSQ Score by Food Type (All) *n*=75 | SSQ Score by Food Type (SSQ<74) *n*=34 | SSQ Score by Food Type (SSQ≥74) *n*=41 | *P* |
|---|---|---|---|---|
| 1. Breads | 15 (15-15) | 15 (0-15) | 15 (15-15)* | 0.01 |
| 2. Spreads | 4.5 (2-7) | 4 (0.38-6) | 5.5 (4-7.3)* | 0.01 |
| 3. Cereals, biscuits and baking | 6 (4-8) | 5.8 (4-8.3) | 6.5 (5-8) | 0.39 |
| 4. Cheeses and Savory Snacks | 7 (5-8.5) | 5 (4-7.6) | 8 (6-9.5)* | <0.01 |
| 5. Tinned and packet foods | 2 (0.5-4) | 1.25 (0-3) | 3 (1-4.5)* | 0.03 |
| 6. Processed meats and seafood | 12 (2-20) | 6.5 (1.5-12.3) | 20 (10-22)* | <0.01 |
| 7. Flavorings added in cooking | 15 (7-21) | 7.5 (1.8-12.8) | 21 (14.5-25)* | <0.01 |
| 8. Flavorings added at the table | 7 (3.5-10) | 4.5 (2.9-7) | 9 (6-13.8)* | <0.01 |
| 9. Pre-prepared meals and drinks | 5 (2-6) | 3.8 (1.6-5) | 5 (4-7.8)* | <0.01 |
| Total SSQ Score | 77 (58-90) | 55.8 (42-67) | 89 (81-94.8)* | <0.01 |

Abbreviations: SSQ, scored salt questionnaire; Data expressed as median values with interquartile ranges in brackets; P value comparing SSQ≥74 vs. SSQ<74 and asterisks denotes $p$ <0.05.

**Table S3.** Median relative percentages of each of the nine sodium-rich food types from the SSQ.

| **Sodium-Rich Food Groups** | **Percentage Contribution (All)** ***n*=75** | **Percentage Contribution (SSQ<74)** ***n*=34** | **Percentage Contribution (SSQ≥74)** ***n*=41** | ***P*** |
|---|---|---|---|---|
| 1. Breads | 19.0 (15.3-25.4) | 22.4 (0-32.1) | 17.4 (15.9-19.5)* | 0.03 |
| 2. Spreads | 6.3 (4.0-8.5) | 6.5 (2.2-10.1) | 6.3 (4.2-7.4) | 0.44 |
| 3. Cereals, biscuits and baking | 8.2 (6.1-12.7) | 11.6 (7.2-15.8) | 7.4 (6-9.1)* | <0.01 |
| 4. Cheeses and Savory Snacks | 9.1 (7.1-12.2) | 11.8 (7.3-15.2) | 8.7 (7.0-10.3)* | <0.05 |
| 5. Tinned and packet foods | 3.4 (0.9-5.4) | 3.2 (0-6.9) | 3.5 (1.3-4.9) | 0.86 |
| 6. Processed meats and seafood | 17.0 (4.9-25.5) | 12.0 (2.1-21.2) | 20.4 (12.9-25.7)* | 0.02 |
| 7. Flavorings added in cooking | 19.5 (9.9-26.7) | 12.2 (3.6-20.6) | 22.3 (15.5-27.3)* | <0.01 |
| 8. Flavorings added at the table | 9.4 (6-14.6) | 8.9 (4.2-14.6) | 9.4 (6.7-14.4) | 0.39 |
| 9. Pre-prepared meals and drinks | 6.2 (3.6-8.5) | 6.4 (2.4-8.6) | 5.8 (4.8-8.3) | 0.98 |

Abbreviations: SSQ, scored salt questionnaire; Data expressed as median values with interquartile ranges in brackets; P value comparing SSQ≥74 vs. SSQ<74 and asterisks denotes $p < 0.05$.
